# Elucidation of relaxin-3 binding interactions in the extracellular loops of RXFP3

**DOI:** 10.3389/fendo.2013.00013

**Published:** 2013-02-22

**Authors:** Ross A. D. Bathgate, Maria H. Y. Oh, W. J. Jason Ling, Quentin Kaas, M. Akhter Hossain, Paul R. Gooley, K. Johan Rosengren

**Affiliations:** ^1^Florey Institute of Neuroscience and Mental Health, University of MelbourneParkville, VIC, Australia; ^2^Department of Biochemistry and Molecular Biology, University of MelbourneParkville, VIC, Australia; ^3^Institute for Molecular Bioscience, The University of QueenslandBrisbane, QLD, Australia; ^4^School of Chemistry, University of MelbourneParkville, VIC, Australia; ^5^School of Biomedical Sciences, The University of QueenslandBrisbane, QLD, Australia

**Keywords:** relaxin-3, RXFP3, peptide, GPCR, modeling

## Abstract

Relaxin-3 is a highly conserved neuropeptide in vertebrate species and binds to the Class A G protein-coupled receptor (GPCR) RXFP3. Relaxin-3 is involved in a wide range of behaviors, including feeding, stress responses, arousal, and cognitive processes and therefore targeting of RXFP3 may be relevant for a range of neurological diseases. Structural knowledge of RXFP3 and its interaction with relaxin-3 would both increase our understanding of ligand recognition in GPCRs that respond to protein ligands and enable acceleration of the design of drug leads. In this study we have used comparative sequence analysis, molecular modeling and receptor mutagenesis to investigate the binding site of the native ligand human relaxin-3 (H3 relaxin) on the human RXFP3 receptor. Previous structure function studies have demonstrated that arginine residues in the H3 relaxin B-chain are critical for binding interactions with the receptor extracellular loops and/or N-terminal domain. Hence we have concentrated on determining the ligand interacting sites in these domains and have focused on glutamic (E) and aspartic acid (D) residues in these regions that may form electrostatic interactions with these critical arginine residues. Conserved D/E residues identified from vertebrate species multiple sequence alignments were mutated to Ala in human RXFP3 to test the effect of loss of amino acid side chain on receptor binding using a Eu-labeled relaxin-3 agonist. Finally data from mutagenesis experiments have been used in ligand docking simulations to a homology model of human RXFP3 based on the peptide-bound chemokine receptor 4 (CXCR4) structure. These studies have resulted in a model of the relaxin-3 interaction with RXFP3 which will inform further interrogation of the agonist binding site.

## INTRODUCTION

Relaxin-3 is a member of the relaxin peptide family. Peptides from this family activate relaxin family peptide (RXFP) receptors, which are Type I or Class A G protein-coupled receptors (GPCRs). It is now well-established that RXFP3 [also called somatostatin- and angiotensin-like peptide receptor (SALPR) or GPCR135] is the endogenous receptor for relaxin-3 (Liu et al., 2003b), although relaxin-3 is also able to bind to and activate both RXFP4 (Liu et al., 2003a) and RXFP1 (Sudo et al., 2003), the receptors of the related insulin-like peptide-5 (INSL5) and relaxin-2, respectively. The relaxin-3 gene is the ancestral gene of the relaxin peptide family (Bathgate et al., 2002) and highly homologous relaxin-3 genes are found in fish, chicken, and frog genomes (Wilkinson et al., 2005b). Relaxin-3 is found in a few discrete nuclei in the brainstem of macaque monkeys (Ma et al., 2009b), rodents (Bathgate et al., 2002; Burazin et al., 2002), and zebrafish (Donizetti et al., 2008) with the most prominent expression in the “nucleus incertus” (NI; Tanaka et al., 2005). This lies in the dorsomedial pons, adjacent to other nuclei involved in neurohumoral responses to stress (Ma et al., 2007). Relaxin-3 neurons project to a number of brain regions where the peptide is localized in presynaptic vesicles of nerve terminals innervating a range of areas containing RXFP3-positive neurons (Ma et al., 2007). These innervated areas are involved in regulating behaviors such as sleep/wakefulness, arousal/attention and mood, stress responses and associated cognitive processes, the activity of which is perturbed in a range of psychiatric diseases and in animal models of these disorders (Ma et al., 2007).

Relaxin-3 is involved in a wide range of behaviors, including feeding, stress responses, arousal, and cognitive processes (Smith et al., 2011). Central administration of relaxin-3 has been shown to increase feeding in rats (McGowan et al., 2005, 2006; Ganella et al., 2012b). A more recent study has demonstrated that chronic viral mediated delivery of a relaxin-3 peptide agonist in the hypothalamus chronically increases feeding and body weight in rats by a novel mechanism that does not make the animals obese (Ganella et al., 2012a). Relaxin-3 neuronal activity and production of relaxin-3 mRNA is increased by stress (Tanaka et al., 2005). RXFP3 antagonist injection into the medial septum inhibits spatial memory in rats, indicating a role for relaxin-3 in cognition (Ma et al., 2009a). Hence targeting of RXFP3 may be relevant for a range of neurological diseases.

**FIGURE 1:**
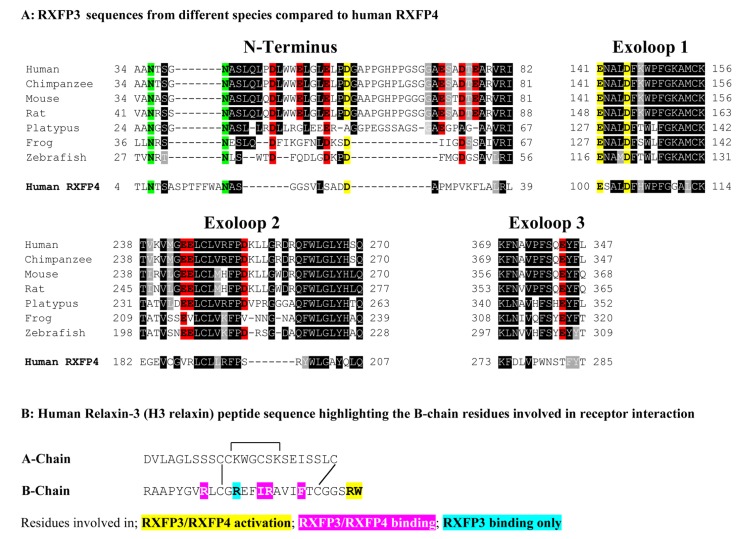
**(A)** Sequence alignment using Boxshade of representative mammalian (human, chimpanzee, rat, mouse, platypus) and representative lower vertebrate RXFP3 sequences (Frog and Zebrafish) compared to human RXFP4. Only residues from the extracellular N-terminus and extracellular loops (ELs) are shown and the N-terminus is aligned from amino acid 34 of human RXFP3. All the glutamic (E) and aspartic acid (D) residues in the extracellular domain are highlighted with those conserved in RXFP3 and RXFP4 in yellow and those conserved in RXFP3 only in red. Conserved potential N-glycosylation sites are highlighted in green. Highly conserved amino acids across all species are boxed in black and conservative amino acid substitutions are boxed and shaded. **(B)** Human relaxin-3 peptide sequence with the key B-chain residues involved in receptor binding and activation highlighted.

The relaxin ligands are complex molecules that are structurally related to insulin comprising two-peptide chains (A- and B-chain) that are cross-braced by three disulfide bonds (Bathgate et al., 2006). The nuclear magnetic resonance solution structure of human relaxin-3 (H3 relaxin) has revealed that the 24 residue A-chain forms two helical segments arranged in an antiparallel fashion, with the 27 residue B-chain adopting a third helix that lies perpendicular to the A-chain helices (Rosengren et al., 2006). Importantly, while features of both the relaxin-3 A- and B-chain are required for binding to RXFP1 (Haugaard-Jonsson et al., 2008; Hossain et al., 2008), the B-chain alone is responsible for interacting with RXFP3 and RXFP4 (Kuei et al., 2007; Hossain et al., 2008; Haugaard-Kedstrom et al., 2011). The A-chain likely provides a scaffold that supports the correct structure of the B-chain (Hossain et al., 2008). Studies using H3 relaxin peptide mutants have demonstrated that residues around the B-chain central helix including R8, R12, I15, R16, and F20 are important for RXFP3 binding whereas all except R12 are also important for RXFP4 binding (Kuei et al., 2007; **Figure 1B**). Additionally, the C-terminal two residues of the B-chain R26 and W27 were demonstrated to be essential for receptor activation (Kuei et al., 2007; **Figure 1B**). It is likely that the residues from the central helix are involved with interactions with the extracellular domains of the receptors while R26 and W27 may interact within the RXFP3 transmembrane helices (Kuei et al., 2007; Kong et al., 2010).

Although residues in the peptides have been identified as critical for activity, the specific residues in the receptors that are interacting with these are unknown. A recent study utilized the selectivity of INSL5 for RXFP4 over RXFP3 and characterized the ligand interactions of chimeric RXFP3/4 receptors in order to identify the N-terminus (NT) and transmembrane exoloop 2 (EL2) of RXFP4 as the sites of INSL5 binding. In addition they determined that sites in transmembrane (TM) 2, 3, and 5 are involved in receptor activation by INSL5 (Zhu et al., 2008). None of the receptor chimeras resulted in appreciable changes in relaxin-3 binding or activity highlighting that relaxin-3 binds to and activates RXFP4 in a similar manner to RXFP3. In this study we have utilized comprehensive sequence analysis of RXFP3 and RXFP4 from multiple vertebrate species to predict interacting residues, focusing on Asp and Glu residues as likely partners for the three key Arg residues in H3 relaxin, and analyzed the effect of mutation of these residues using binding studies. Finally ligand docking and molecular modeling was used to determine potential sites of interaction of relaxin-3 with the RXFP3 receptor.

## MATERIALS AND METHODS

### REAGENTS

Synthetic H3 relaxin was chemically prepared as previously described (Bathgate et al., 2006). The mono-Eu-labeled relaxin-3 agonist H3 relaxin B-chain, INSL5 A chain (H3/I5) which maintained high affinity for receptor binding studies, has been described previously (Luo et al., 2010). All synthetic oligonucleotides were purchased from Sigma Aldrich and are listed in **Tables 1 and 2**. PCR reactions and cloning were undertaken as previously described (Scott et al., 2006, 2012).

**Table 1:** Primers for the production of pcDNA3.1 (+)-HA, pcDNA3.1 (+)-HA-RXFP3, and pcDNA3.1 (+)-HA-RXFP3 ∆1–33 constructs.

Construct	Primer name	Sequence (5′–3′)
pcDNA3.1 (+)-HA	HA Tag Fwd	GAT CCG CCA CCA TGT ACC CAT ACG ATG TTC CAG ATT ACG CTG AT
	HA Tag Rev	ATC AGC GTA ATC TGG AAC ATC GTA TGG GTA CAT GGT GGC G
pcDNA3.1 (+)-RXFP3-HA	RXFP3-HA *Eco*RV (no ATG) Fwd	GAG AGG ATA TCC AGA TGG CCG ATG CAG CCA C
	RXFP3-HA Rev	GAG AGC TCG AGT CAG TAG GCA GAG CTG CTG G
pcDNA3.1(+)-RXFP3-HA δ1–33	*Eco*RV Fwd	GAGAG GAT ATC GCG GCC AAC ACG AGT GG
	pcDNAMCS Rev	CAA CAG ATG GCT GGC AAC TA

**Table 2:** Site-directed mutagenesis primers for HA-RXFP3 D/E to A mutants.

Mutation	Direction	Primer sequence (5′–3′)
E47A/D51A	Fwd	CCG GCC TTG TGG TGG GCG CTG GGG C
	Rev	CAG CGC CCA CCA CAA GCC CGG AAG CTG
E55A/D58A	Fwd	CTG GCC TTG CCG GCC GGC GCG CCG CC
	Rev	GCC GGC CGG CAA GGC CAG CCC CAG CTC CCA CC
E72A	Fwd	GCA GCG AGC GCG GAC ACA GAG GCC C
	Rev	GCT CGC TGC CCC GCC GCT G
D75A	Fwd	GCG GCC ACA GAG GCC CGG GTG C
	Rev	C TGT GGC CGC GCT CTC TGC CCC
E77A	Fwd	CACA GCG GCC CGG GTG CGG ATT CTC ATC
	Rev	GGC CGC TGT GTC CGC GCT CTC TGC CC
E141A/D145A	Fwd	GTG GCC AAC GCT CTT GCC TTC AAA TGG CCC TTC GGC AAG G
	Rev	GAA GGC AAG AGC GTT GGC CAC CGC CCA GAA GGG CAG
E141A	Fwd	GTG GCC AAC GCT CTT GAC TTC AAA TGG CCC TTC
	Rev	GTT GGC CAC CGC CCA GAA GGG CAG
D145A	Fwd	CTT GCC TTC AAA TGG CCC TTC GGC AAG GCC
	Rev	GAA GGC AAG AGC GTT CTC CAC CGC CCA G
E244A	Fwd	GGC GCC GAG CTG TGC CTG GTG CGT TTC
	Rev	CTC GGC GCC CAT CAC CTT GAC CGT GG
E245A	Fwd	GAG GCC CTG TGC CTG GTG CGT TTC C
	Rev	CAG GGC CTC GCC CAT CAC CTT GAC CG
E253A	Fwd	CCG GCC AAG TTG CTG GGC CGC G
	Rev	CTT GGC CGG GAA ACG CAC CAG GC
D259A	Fwd	CGC GCC AGG CAG TTC TGG CTG GGC CTC
	Rev	CCT GGC GCG GCC CAG CAA CTT GTC CG
E362A	Fwd	CAG GCG ATT TTCC TGT GCC AGG TAT ACG CGT TC
	Rev	GAA ATA CGC CTG GCT GAA GGG CAC CGC G

### RXFP3 SEQUENCE ANALYSIS

To highlight potential ligand interacting residues in the RXFP3 extracellular domains, we performed multiple sequence alignments of all the RXFP3/4 sequences identified from mammalian genomes together with RXFP3/4 sequences from lower vertebrate genomes (Wilkinson et al., 2005a; Wilkinson and Bathgate, 2007). Sequences were retrieved from available genomes at Ensembl (http://www.ensembl.org) and NCBI and aligned using ClustalW with default parameters and shaded using Boxshade.

### MAMMALIAN EXPRESSION CONSTRUCTS

A pcDNA3.1zeo (+) vector with a start codon and HA tag (pcDNA3.1zeo (+)-HA) was produced by ligating a double stranded primer containing a *Bam*HI site at the 5′ end followed by a start codon and HA tag and then a *Eco*RV blunt site at the 3′ end (**Table 2**) into a pcDNA3.1zeo (+) vector cut with *Bam*HI and *Eco*RV. The 1XHA-tagged human RXFP3 vector (pcDNA3.1zeo (+)-HA-RXFP3) was produced by ligating a polymerase chain reaction (PCR) generated RXFP3 construct missing the ATG start codon and with a *Eco*RV site at the 5′ end and a *Xho*I site at the 3′ end following the TGA stop site (primers listed in **Table 2**) into the pcDNA3.1zeo (+)-HA construct. The DNA template of N-terminal truncated mutant (HA-RXFP3 ∆1–33) was prepared by PCR amplification from pcDNA3.1zeo (+)-HA-RXFP3 using forward and reverse primers containing *Eco*RV and *Xho*I sites, respectively (listed in **Table 1**). This product was ligated in the pcDNA3.1zeo (+)-HA construct to create pcDNA3.1zeo (+)-HA-RXFP3 ∆1–33. Final constructs were confirmed by DNA sequencing on both strands while at the same time confirming that there were no additional unwanted mutations in the sequence of the receptor.

### SITE DIRECTED MUTAGENESIS

The same protocol was used to prepare DNA templates of all Glu/Asp to Ala mutants, entailing a single codon change except for D47A/E51A, E55A/D58A, and E141A/D145A which required a double codon change both to Ala. The forward and reverse primers for all mutants are listed in **Table 1** and mutagenesis reactions on pcDNA3.1zeo (+)-HA-RXFP3 were performed as previously described (Scott et al., 2006; Yan et al., 2008). Individual clones were screened, and the identities of individual mutations were confirmed by DNA sequencing on both strands while at the same time confirming that there were no additional unwanted mutations in the full length sequence of the receptor.

### LIGAND BINDING AND CELL SURFACE EXPRESSION ASSAYS

HEK-293T cells were transfected in 96 well optiplates (Perkin Elmer) with plasmids encoding the constructs of interest and Eu-H3/I5 binding assays conducted as described previously (Luo et al., 2010; Haugaard-Kedstrom et al., 2011). Specific binding assays were conducted using 5 nM Eu-H3/I5 in the absence (total binding) or presence (non-specific binding) of 1 μM unlabeled H3 relaxin to determine specific binding (total binding - non-specific binding). Competition binding assays were performed as above with increasing concentrations of unlabeled H3 relaxin and non-specific binding was determined using 1 μM unlabeled H3 relaxin. Binding data are expressed as mean ± standard error of mean (SEM) of % specific binding of triplicate measurements pooled from at least three independent experiments. Data were analyzed using Graphpad PRISM (Graphpad Inc.) and a non-linear regression one-site binding model was used to plot curves and calculate pIC_50_ values. All the RXFP3 constructs used in this study contained an N-terminal HA epitope. The HA epitope was detected using a purified mouse monoclonal anti-HA antibody [HA.11(16B12), Covance] and a Alexafluor488-labeled goat anti mouse IgG (Invitrogen) in cell surface expression assays that were performed in 24 well plates as previously described (Yan et al., 2008). Cell surface expression was determined by subtracting the non-specific binding in pcDNA3.1 control transfected cells and then dividing by the HA-RXFP3 expression to give %HA-RXFP3 expression. Specific binding data for RXFP3 mutants was expressed as mean ± SEM of % specific binding/%HA-RXFP3 cell surface expression of triplicate measurements pooled from at least three independent experiments. Pooled data were analyzed using one-way ANOVA coupled to Newman–Keuls multiple comparison test for multiple group comparison.

### MOLECULAR MODELING

A model of RXFP3 in complex with H3 relaxin was created using the NMR solution structure of H3 relaxin (PDB identifier 2fhw) (Rosengren et al., 2006) and the crystal structure of the chemokine receptor CXCR4 in complex with the antagonist CVX15 (PDB identifier 3oe0; Wu et al., 2010). An alignment between the sequences of the two receptors was initially created using Muscle (Edgar, 2004). CXCR4 shares 30% identity and 60% similarity with RXFP3. RXFP3 has 80 additional residues at the N-terminus compared to the crystal structure of CXCR4, and this part of the protein has no homologous structure in the PDB. Consequently, the first 50 residues of RXFP3 were not modeled, whereas the remaining part of the N-terminus, which has been shown to have some influence on the activity, was modeled as disordered. Extracellular loops 1 and 2 comprise two and seven additional residues, respectively, in RXFP3 compared to CXCR4. The additional residues were inserted at positions 101–102 and 192–193 (CXCR4 numbering). A model of RXFP3 was build by comparison using Modeller 9v10 (Sali and Blundell, 1993), and the two loops that are longer in RXFP3 were further refined within Modeller (Fiser et al., 2000). H3 relaxin was docked into the receptor during the comparative modeling procedure using the similarity of sequence between the N-terminus of CVX15 and of the C-terminus of the H3 relaxin chain B, as well as by introducing loose distance restraints between the negatively charged patch on the surface of RXFP3 that was shown to be important for binding (D145 and E244) and the positively charged residues R12 and R16 of the H3 relaxin B-chain. The two residues R2 and naphthalene-2-yl-3-alanine at position 3 of CVX15 deeply dive into the binding site in between the transmembrane helices of CXCR4, and their physico–chemical similarities with the B-chain residues R26 and W27, which are vital for activity of H3 relaxin, were used to introduce positional restraints between H3 relaxin and RXFP3. 100 models were generated using Modeller and the model with the lowest discrete optimized protein energy (DOPE) scope (Shen and Sali, 2006) was selected as the most representative.

## RESULTS

### SEQUENCE ALIGNMENTS

**FIGURE 2:**
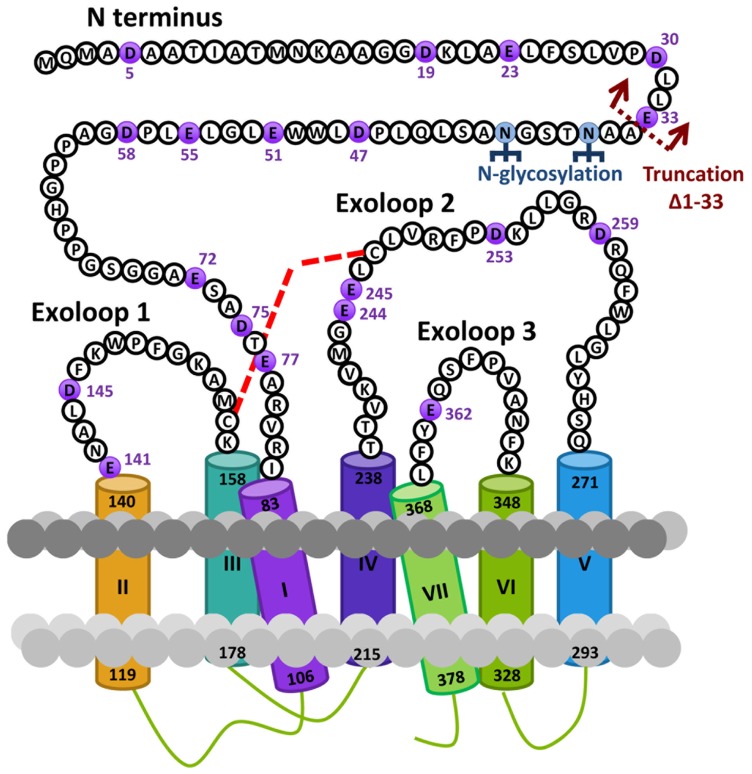
**Snake diagram of the extracellular domains of the human RXFP3 receptor highlighting residues from the N-terminus and extracellular loops (ELs)**. The glutamic (E) and aspartic acid (D) residues in these domains that have been mutated to alanine in this study are highlighted in purple. The site of truncation to form the N-terminally truncated receptor HA-RXFP3 ∆1–33 is indicated and potential N-gylcosylation sites are also highlighted. The putative disulphide bond between C156 in EL1 and C247 in EL2 is represented with a red dotted line.

Based on the hypothesis that coevolution of relaxin-3 and RXFP3 occurred in vertebrates and that residues important for relaxin-3 binding and activation should therefore be conserved we performed multiple sequence alignments of all vertebrate RXFP3 sequences. As H3 relaxin also binds to and activates human RXFP4 (Liu et al., 2003a) we have also used human RXFP4 in the sequence analysis. **Figure 1A** highlights sequence alignments of representative mammalian species (human, chimpanzee, rat, mouse, platypus) and representative lower vertebrate species (Frog and Zebrafish) from this analysis compared to human RXFP4. We have concentrated on the extracellular domains of the receptor as residues in the N-terminus and ELs are likely interacting with the key binding residues in the central helix of the relaxin-3 B-chain (**Figure 1B**). Additionally, we have focused on glutamic (E) and aspartic acid (D) residues in these regions that may form electrostatic interactions with critical arginine residues in the relaxin-3 B-chain. As R12 has been demonstrated to not be involved in RXFP4 binding whereas R8 and R16 have (Kuei et al., 2007), we have highlighted residues conserved in only RXFP3 sequences in red and those conserved in RXFP3 and RXFP4 in yellow. All of these identified E/D residues are highlighted in more detail in **Figure 2** which also demonstrates where we have truncated the N-terminus to residue 34 to produce the mutant HA-RXFP3 ∆1–33. This construct removes the D5, D19, E23, D30, and E33 residues while keeping the highly conserved potential N-glycosylation sites, which are likely to be essential for receptor cell surface expression (**Figure 2**).

### EFFECTS OF MUTAGENESIS ON BINDING

**FIGURE 3:**
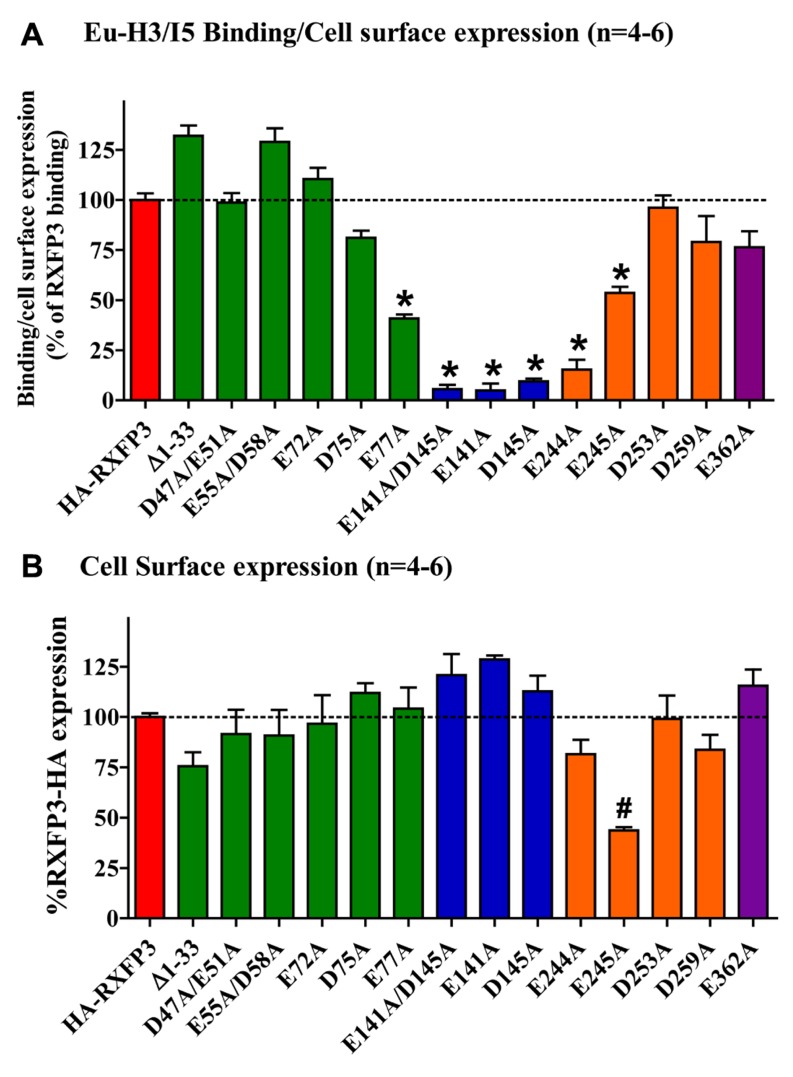
**(A)** Eu-H3/INSL5 binding to mutant receptors expressed on the surface of cells. Data are expressed as a ratio of specific Eu-H3/I5 binding to the cell surface expression (**B**) of each construct and are normalized to HA-RXFP3 binding. **(B)** Cell surface expression of the various RXFP3 mutant receptors compared to HA-RXFP3. All data are the mean of triplicate determination from 4 to 8 independent experiments. *p < 0.001 vs HA-RXFP3, #*p* < 0.01 vs HA-RXFP3.

Conserved E/D residues identified from sequence alignments were mutated to Ala in human RXFP3 (highlighted in **Figures 1 and 2**) to test the effect of loss of amino acid side chain on receptor binding using the Eu-labeled RXFP3 agonist H3/I5. Receptor mutants were tested in parallel for their cell surface expression and final specific binding data was expressed as a ratio of binding/cell surface expression (**Figure 3**). Due to their close proximity D47A/E51A, E55A/D58A, and E141A/D145A were made as combination mutants in the first instance to test the effect of the loss of both side-chains. Importantly it was immediately clear that the mutation of E141A/D145A resulted in a dramatic loss of binding with little effect on the cell surface expression of the mutant receptors. We therefore produced the individual receptor mutants E141A and D145A and demonstrated that both of these mutations resulted in a dramatic decrease in binding with no change in cell surface expression. In contrast, the mutants D47A/E51A demonstrated no loss in binding whereas E55A/D58A demonstrated a slight increase in binding and were therefore not followed up with individual mutations. All of the other identified D/E residues, E72A, D75A, E77A, E244A, E245A, D253A, E362A were produced and tested with most showing no change in binding or cell surface expression. However, the EL2 residues which are in close proximity E244A and E245A demonstrated significant loses in binding with E245A also showing significantly lower cell surface expression. Additionally, E77A showed a ~50% loss of binding with no change in cell surface expression. Interestingly the truncated mutant ∆1–33 demonstrated a slight increase in binding demonstrating that D5, D19, E23, D30, and E33 residues are unlikely to be involved in H3 relaxin binding.

**FIGURE 4:**
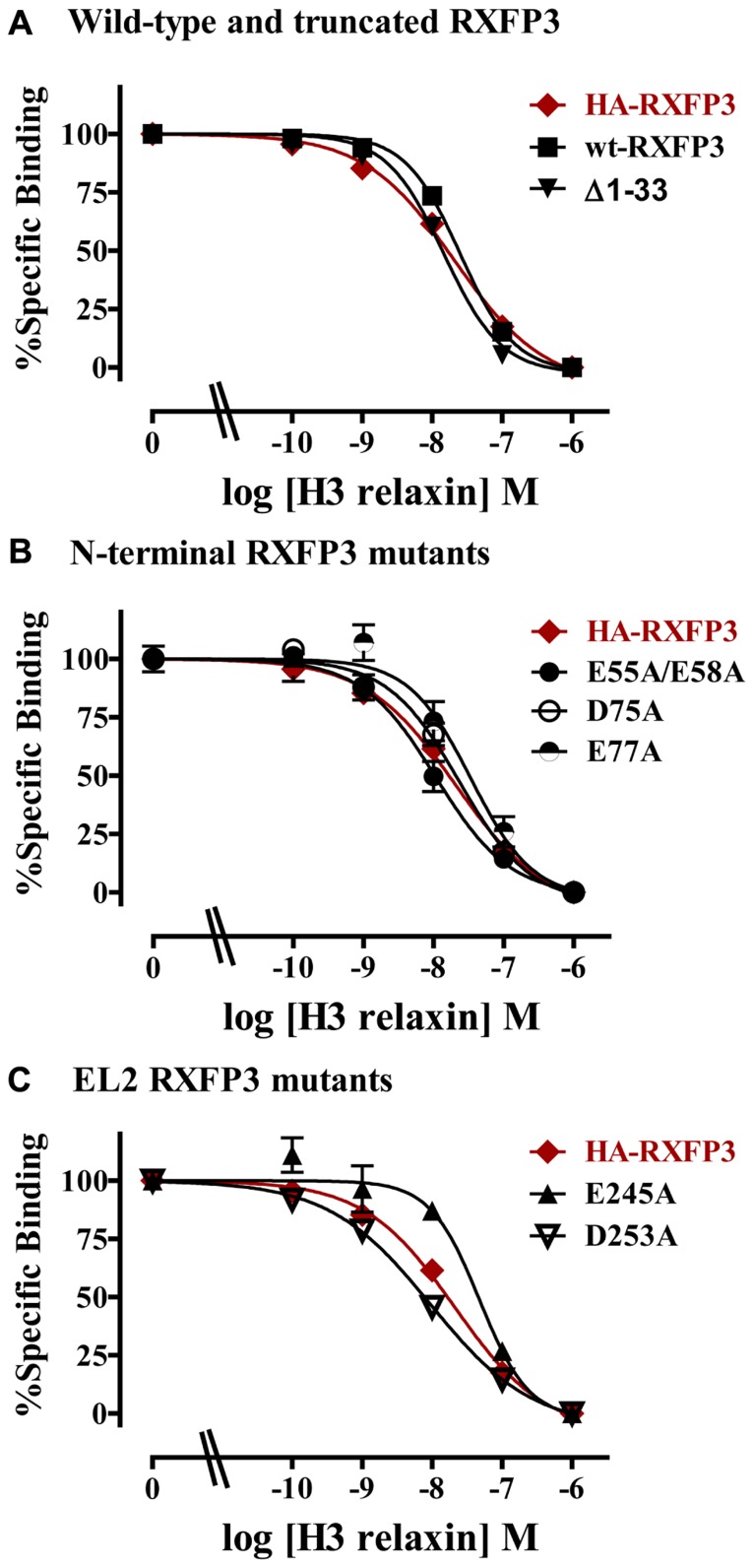
**Competition binding data for representative mutant RXFP3 receptors compared to HA-RXFP3**. **(A)** Wild-type RXFP3 and the N-terminally truncated mutant ∆1–33. **(B)** N-terminal domain mutants. **(C)** Exoloop 2 mutants. Data are presented as pooled % specific binding of triplicate determinations from 4 to 9 independent experiments.

The specific binding data clearly identified E141A and D145A in EL1 and E244A in EL2 as being involved in H3 relaxin binding, with possible additional contributions from E77A in the N-terminus and E245 in EL2. The specific binding levels of E141A, D145A, and E244A were too low for further analysis. However, both E77A and E245A demonstrated enough specific binding to enable further analysis using competition binding assays using H3 relaxin as the competing ligand. They were therefore compared with wild-type RXFP3, HA-RXFP3 and various other mutants which demonstrated either no change in (D75A), or slightly increased (∆1–33, E55A/D58A), specific binding (**Figure 4**; **Table 3**). Importantly as can be seen in **Figure 4** and in **Table 3** there was no difference in the binding affinity of H3 relaxin to HA-RXFP3 in comparison to wild-type RXFP3 (pIC50 = 7.53 ± 0.06 and 7.89 ± 0.14, respectively). The truncation of the N-terminus to produce ∆1–33 also had no significant effect on the affinity of H3 relaxin (pIC50 = 7.83 ± 0.08) although there was a slightly higher specific binding of Eu-H3/I5. There was also no significant difference in the affinity of H3 relaxin for D75A (pIC50 = 7.70 ± 0.16) and although the affinity of E55A/D58A was slightly higher in line with the higher Eu-H3/I5 binding, this was not significantly different from HA-RXFP3 (pIC50 = 8.03 ± 0.20, *p*> 0.05). Finally both E77A and E245A which demonstrated an ~50% decrease in Eu-H3/I5 binding demonstrated slightly lower H3 relaxin binding affinity but this was not significantly different from HA-RXFP3 (pIC50 = 7.50 ± 0.22 and 7.35 ± 0.09, respectively, *p*> 0.05).

**Table 3:** Competition binding results for HA-RXFP3 mutants.

Construct	pIC_50_	*n*
RXFP3	7.53 ± 0.06	9
HA-RXFP3	7.89 ± 0.14	8
HA-RXFP3 ∆1–33	7.83 ± 0.08	4
HA-RXFP3 E55A/D58A	8.03 ± 0.20	4
HA-RXFP3 D75A	7.70 ± 0.16	4
HA-RXFP3 E77A	7.50 ± 0.22	4
HA-RXFP3 245A	7.35 ± 0.09	4

### MODEL OF RELAXIN-3/RXFP3 INTERACTION

To illustrate the nature of H3 relaxin–RXFP3 complex the mutational data on RXFP3 generated in this study and previously reported structure activity relationships of H3 relaxin were used to generate a molecular model. As starting structures we used the NMR solution structure of H3 relaxin (Rosengren et al., 2006) and the crystal structure of the chemokine receptor CXCR4 in complex with the antagonist CVX15 (Wu et al., 2010). Although the structure of CXCR4 is in its inactive state this structure represents a good template for modeling the binding site given it is closely related to RXFP3 (30% identity and 60% similarity) and it is bound to a peptide ligand creating a more “open” conformation required for binding of a large ligand like H3 relaxin. The sequences of the seven transmembrane helices as well as some of the loop regions could be aligned between receptors without ambiguity, allowing a homology model of RXFP3 to be built by comparison using the program package Modeller 9v10 (Sali and Blundell, 1993). H3 relaxin was docked into the receptor during the comparative modeling procedure by utilizing the similarities between key features of the CVX15 ligand and the activation domain of H3 relaxin, R26 and W27, as well as loose distance restraints bringing the positively charged key arginine residues of the ligand, R12 and R16, into the proximity of the key negatively charged residues D145 and E244 of RXFP3.

**FIGURE 5:**
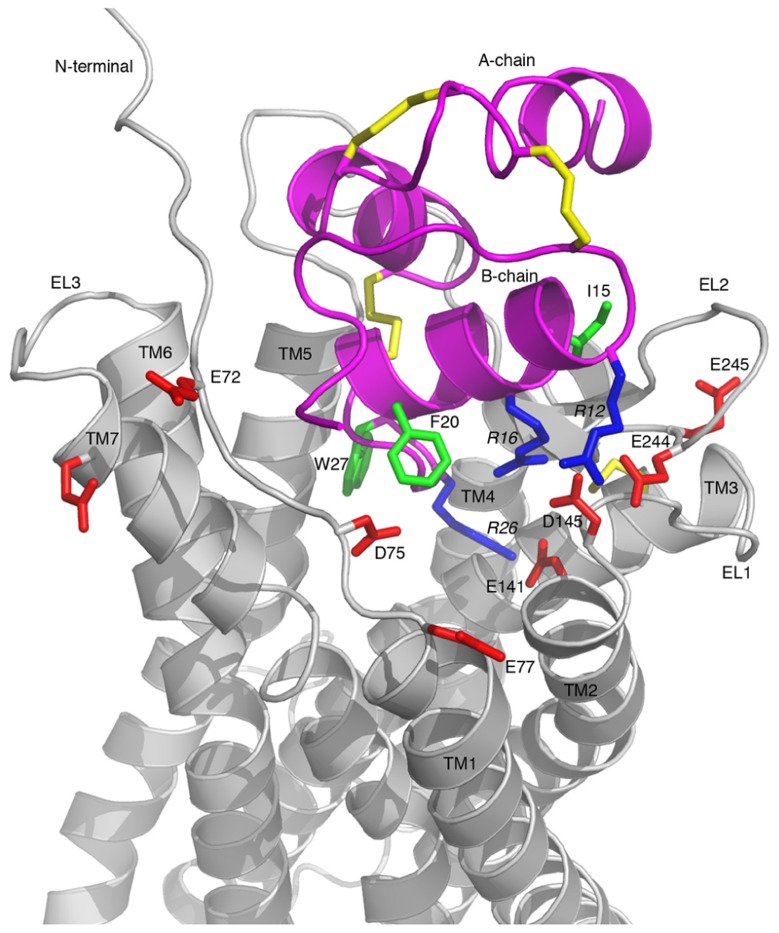
**Model of the RXFP3-H3 relaxin complex**. H3 relaxin is shown in pink and RXFP3 in gray. All Asp/Glu residues in RXFP3 mutated during this study are shown in red and labeled with residue numbers. Key Arg and hydrophobic residues in H3 relaxin involved in the receptor binding are highlighted in blue and green, respectively, and labeled with residue numbers. Key features are the electrostatic interactions between the positively charged R12, R16, and R26 of H3 relaxin and the negatively charged E244, D145, and E141 of RXFP3, respectively.

A representative model of the complex is shown in **Figure 5**, which highlights the key features of the proposed binding mode. The binding surface of the H3 relaxin B-chain helix packing up against EL1 and EL2, creating a network of electrostatic interactions between R16 and D145 as well as between R12 and E244. This arrangement suggests that the more hydrophobic face of the helix, which includes I15 and I19 is able to interact with a hydrophobic region on EL2. The C-terminus of the H3 relaxin B-chain is able to be accommodated deep into the transmembrane helical bundle with R26 directly interacting with the critical E141 at the top of TM2, consistent with this region being able to induce the structural rearrangement required for activation. No direct contacts with the ligand are observed, or likely to be possible by minor rearrangements, for either E77 or E245. Thus the minor effects of mutations observed for these residues are likely related to minor destabilization of the fold.

## DISCUSSION

Recent physiological evidence has highlighted an important role of the relaxin-3/RXFP3 signaling system in a number of neurological signaling processes including feeding, stress responses, arousal, and cognition (Smith et al., 2011; Ganella et al., 2012b). These findings have sparked considerable interest in RXFP3 as a potential pharmacological target for multiple neurological diseases. However, the only current agonists or antagonists that are known to target this receptor are peptides which must be administered intracerebroventricularly for *in vivo* studies in rodents (Haugaard-Kedstrom et al., 2011; Shabanpoor et al., 2012). Although residues in these peptides have been identified as critical for activity, the specific residues in the receptors that are interacting with these are unknown. Detailed knowledge of relaxin-3 binding site in RXFP3 would enable a structure-based drug design approach to design small molecule agonists and antagonists. We have therefore used comparative sequence analysis, molecular modeling and receptor mutagenesis to investigate the binding site of the native ligand H3 relaxin on the human RXFP3 receptor.

Previous peptide structure function studies have demonstrated that the H3 relaxin B-chain alone is interacting with the receptor (Liu et al., 2003b; Hossain et al., 2008). Subsequently it has been demonstrated that residues around the H3 relaxin B-chain central helix including R8, R12, I15, R16, and F20 are important for RXFP3 binding whereas all except R12 are also important for RXFP4 binding (Kuei et al., 2007). Additionally, the C-terminal two residues of the B-chain R26 and W27 were demonstrated to be essential for receptor activation (Kuei et al., 2007; **Figure 1B**). The evidence suggests that the H3 relaxin B-chain is binding to the extracellular domains of the receptor using residues from the central helix whereas R26 and W27 may interact within the RXFP3 transmembrane helices to induce the active receptor conformation (Kuei et al., 2007; Kong et al., 2010). So in this study we have focused our attention on the binding interactions between the residues in the central helix of the H3 relaxin B-chain and the RXFP3 ELs and/or NT domain.

Based on the hypothesis that coevolution of relaxin-3 and RXFP3 occurred in vertebrates, and in the knowledge that the key relaxin-3 B-chain residues are conserved across all vertebrate species (Wilkinson et al., 2005b), we performed multiple sequence alignments of all vertebrate RXFP3 sequences to identify highly conserved residues in the ELs and NT domain that may be involved in relaxin-3 binding. As H3 relaxin also binds to and activates human RXFP4 (Liu et al., 2003a) we have also used human RXFP4 in the sequence analysis. We have focused on glutamic (E) and aspartic acid (D) residues in these regions that may form electrostatic interactions with critical arginine residues in the relaxin-3 B-chain. As R12 has been demonstrated to not be involved in RXFP4 binding whereas R8 and R16 have (Kuei et al., 2007), it is likely that some E/D residues are conserved in RXFP4 but some not. All of the identified E/D residues are highlighted in **Figure 2** which also demonstrates where we have truncated the N-terminus to residue 34 to produce the mutant HA-RXFP3 ∆1–33. Truncation of the N-terminus is an easy method to exclude residues as potential interacting sites with the ligand. We only truncated RXFP3 to residue 34 as there are highly conserved potential N-glycosylation sites at N36 and N40, which are likely to be essential for receptor cell surface expression as has been demonstrated for numerous GPCRs (Lanctot et al., 2005) including the related RXFP1 receptor (Yan et al., 2008).

Conserved D/E residues in the ELs and NT domain were mutated to Ala in human RXFP3 to test the effect of loss of amino acid side chain on receptor binding using the Eu-labeled relaxin-3 agonist Eu-H3/I5. The specific binding assays identified almost complete loss of binding for residues E141A and D145A in EL1 and residue E244A in EL2. Additionally, E77A in the NT domain and E245A in EL2 demonstrated decreased specific binding although when analyzed with more detailed competition binding there was no significant difference in H3 relaxin affinity. Importantly, the E245A mutant also demonstrated lower cell surface expression indicating that this residue may be important for the EL structure. None of the other D/E mutations showed any significant change in binding or cell surface expression as was the case with the ∆1–33 truncation highlighting that residues 1–33 and other D/E residues are not involved in H3 relaxin binding. It is therefore clear that E141A and D145A in EL1 and residue E244A in EL2 are essential for H3 relaxin binding.

Based on the mutational data a model was created utilizing structural information in the form of a solution NMR structure of the H3 relaxin ligand (Rosengren et al., 2006) and a homology model of RXFP3 derived from the crystal structure of CXCR4 (Wu et al., 2010). Although the structure of CXCR4 represents an antagonist bound inactive form it is a good template for visualizing the binding site as RXFP3 and CXCR4 are closely related and both respond to stimulation by protein ligands, rather than small molecules. For both relaxin-3 and the native ligand of CXRC4, CXCL12, a similar model of receptor binding and activation has been proposed, which involves two steps: first, an initial recognition that involves an interaction between the globular part of the protein ligand and the extracellular loops of the receptor and second, an insertion of a flexible arm into the transmembrane helical bundle to induce a structural change leading to intracellular signaling (Crump et al., 1997). It is interesting to note that in the case of CXCL12 this “activation arm” is located at the N-terminus while for relaxin-3 the activation domain is at the C-terminus of the B-chain.

The model of the RXFP3 receptor reveals that the three acidic residues identified as critical for ligand binding, E141, D145, and E244, are ideally positioned to coordinate three arginines in relaxin-3. R12 and R16, which are located on the B-chain helical segment, interact with E244 and D145, respectively. This arrangement allows the more flexible C-terminal tail of H3 relaxin to insert deeper into the receptor’s binding pocket where R26 can form a salt-bridge with E141, which is located at the C-terminal end of TM2. These pairings of electrostatic residues are consistent with the sequence analysis showing that E141 and D145 are conserved across all RXFP3/4 sequences whereas E244 is only conserved in RXFP3 sequences, thus E244 is likely to be involved in the R12 interaction which is relaxin-3/RXFP3-specific. Although it is difficult to draw too many conclusions about the exact orientation of the activation domain within the TM bundle without further mutational data it is interesting to note that in the suggested arrangement W27 forms extensive hydrophobic contacts with residues at the TM5/TM6 interface, and it is possible that a coordinated effect resulting from this interaction together with the R26–E141 salt-bridge induces the rearrangement of the bundle resulting in activation of the receptor. Furthermore, the negatively charged residue at position 141 is conserved also in CXCR4 (D97). In a CXCR4 crystal structure with a small molecule antagonist bound this Asp forms a salt-bridge with the ligand (Wu et al., 2010), and given it has also been shown to be important for binding of the native agonist ligand CXCL12 (Brelot et al., 2000), this ligand interaction point appears to be conserved between the receptors.

The mutational data also suggested a possible involvement of E77 and E245 in ligand binding, however, neither is in contact with relaxin-3 in our model. Competition binding experiments using H3 relaxin did not show a significant drop in binding to either of these residues thus it seems likely that the reduced specific binding of the Eu-H3/I5 tracer may be related structural effects of these mutations, rather than direct contacts. This suggestion is consistent with the E245A analog having significantly lower surface expression that may be related to misfolding or misprocessing. Of the remaining residues in H3 relaxin identified as contributing to binding I15 and I19 are in close contact with EL2 in our model, while R8 and F20 do not appear to directly contact the receptor in the suggested arrangement. It is however, possible that both these residues could come into contact with the N-terminal domain, should it move closer to the ligand. The exact positioning of the N-terminus is highly ambiguous based on current data, due to low homology and defined structural features it was in this study modeled as disordered. However, studies on chimeric RXFP3/RXFP4 receptor have suggested that in fact it does contribute, at least to the INSL5 binding to RXFP4 (Zhu et al., 2008), thus structural changes that allows such contacts is a possible explanation for this observation.

In summary, we have in this study provided the first information about the ligand binding site of the relaxin-3 receptor RXFP3. We have identified three negatively charged residues in EL1 and EL2 that coordinate an interaction with three positively charged arginines in H3 relaxin. Our data provide a first model of relaxin-3 interaction with RXFP3, which will inform further interrogation of the agonist binding site.

## Conflict of Interest Statement

The authors declare that the research was conducted in the absence of any commercial or financial relationships that could be construed as a potential conflict of interest.
